# Advances in Adoptive Cell Therapy Using Induced Pluripotent Stem Cell-Derived T Cells

**DOI:** 10.3389/fimmu.2021.759558

**Published:** 2021-09-28

**Authors:** Ratchapong Netsrithong, Methichit Wattanapanitch

**Affiliations:** ^1^ Siriraj Center for Regenerative Medicine, Faculty of Medicine Siriraj Hospital, Mahidol University, Bangkok, Thailand; ^2^ Department of Immunology, Faculty of Medicine Siriraj Hospital, Mahidol University, Bangkok, Thailand

**Keywords:** adoptive cell therapy, induced pluripotent stem cells, T cells, chimeric antigen receptor, tumor infiltrating lymphocytes, cancer immunotherapy, off-the-shelf T cells

## Abstract

Adoptive cell therapy (ACT) using chimeric antigen receptor (CAR) T cells holds impressive clinical outcomes especially in patients who are refractory to other kinds of therapy. However, many challenges hinder its clinical applications. For example, patients who undergo chemotherapy usually have an insufficient number of autologous T cells due to lymphopenia. Long-term *ex vivo* expansion can result in T cell exhaustion, which reduces the effector function. There is also a batch-to-batch variation during the manufacturing process, making it difficult to standardize and validate the cell products. In addition, the process is labor-intensive and costly. Generation of universal off-the-shelf CAR T cells, which can be broadly given to any patient, prepared in advance and ready to use, would be ideal and more cost-effective. Human induced pluripotent stem cells (iPSCs) provide a renewable source of cells that can be genetically engineered and differentiated into immune cells with enhanced anti-tumor cytotoxicity. This review describes basic knowledge of T cell biology, applications in ACT, the use of iPSCs as a new source of T cells and current differentiation strategies used to generate T cells as well as recent advances in genome engineering to produce next-generation off-the-shelf T cells with improved effector functions. We also discuss challenges in the field and future perspectives toward the final universal off-the-shelf immunotherapeutic products.

## Introduction

Adoptive cell therapy (ACT) of T lymphocytes offers a potential therapy for chronic viral infection and cancers. ACT can be achieved by isolating T cells from the excised tumor mass (tumor infiltrating lymphocytes or TILs), *ex vivo* expanding and reinfusing them into the patient to target viral or tumor antigens (1, 2). However, the process of TIL isolation and expansion limits their clinical applications since it is technically difficult, labor-intensive, costly and difficult to standardize. TILs do not often provide potent anti-tumor effects due to exhaustion of T cells. In addition, identification of antigen-specific T cells in other solid tumors is very challenging (3). To improve specificity and cytotoxicity of ACT, genetic engineering approaches to target the antigens by transduction of antigen-specific T cell receptor (TCR) or chimeric antigen receptor (CAR) gene can be performed. The engineered T cells are then expanded and reinfused into the patient after lymphodepletion. The TCR-engineered T cells recognize target antigens, which are processed within the cytoplasm and presented by specific human leukocyte antigen (HLA) or major histocompatibility complex (MHC) class I molecules on the surface of the viral-infected cells or cancer cells (4). Several studies reported the use of TCR-engineered T cells to treat patients including NY-ESO-1-directed tTCR and MAGE-A3-directed tTCR for multiple myeloma (MM) (5, 6), and WT1-directed tTCR for acute myeloid leukemia (AML) (7). However, ACT using TCR-engineered T cells is limited by the need to engineer TCR specific for antigen and MHC molecules of the patient.

In contrast, antigen recognition by CAR is mediated by a synthetic hybrid receptor composed of an extracellular antigen-recognition domain, which is a single-chain variable fragment (scFv) derived from the variable regions of a monoclonal antibody (mAb), a transmembrane (TM) domain and intracellular signaling domains such as TCR-derived CD3ζ and co-stimulatory domains (CD28 or 4-1BB) (8). Unlike TCR-engineered T cells, CAR T cells can recognize a specific antigen and eliminate the tumor cells in an HLA-independent manner, therefore, enhancing therapeutic outcomes. Clinical trials using CAR T cell therapy showed a long-term remission in both hematological malignancies and solid tumors (9, 10). To date, the US FDA approved four CD19-directed CAR T cell products: Kymriah™ in 2017 and Yescarta™ in 2018, Tecartus™ in 2020, and recently Breyanzi^®^ in 2021, for the treatment of relapsed or refractory B cell malignancies (1, 2). Despite its remarkable success, ACT using autologous TCR- or CAR-engineered T cells has some unavoidable limitations. The ACT therapy relies on personalized manufacture, which proves very challenging in terms of time and cost to manufacture T cells thereby restrictive for large-scale clinical applications. Moreover, it is also technically difficult to obtain sufficient number of autologous T cells from lymphopenic patients who are heavily pretreated with chemotherapy, or immunodeficient patients, to generate a clinically relevant dose of T cells for therapy (3, 11). In order to obtain sufficient number of cytotoxic T cells (CTLs) for ACT, *ex vivo* expansion to enrich the number of CTLs is required before infusion. This process involves several stimulation steps using various cytokines to increase T cell proliferation. Long-term culture can drive CTLs into an “exhausted state”, where CTLs have shortened telomere length, and lose proliferative capacity and effector function, which hinder their clinical practicality (4, 5).

One way to generate an unlimited supply of universal allogeneic CAR T cells for cancer immunotherapy is to use induced pluripotent stem cells (iPSCs) as a starting material. Advances in iPSC technology have made the generation of autologous pluripotent stem cells (PSCs) possible. These cells have unlimited proliferation and can be differentiated into all specialized cell types of the body; therefore, they represent an autologous renewable cell source for regenerative medicine. iPSCs can be derived from various somatic cell sources, mainly skin fibroblasts and peripheral blood, by introducing the Yamanaka factors (OCT4, SOX2, KLF4 and c-MYC) (6, 7). One of the useful applications of iPSCs in regenerative medicine is the production of CTLs for viral or cancer immunotherapy. Previous studies demonstrated that iPSCs generated from T cells retained rearranged TCR genes. Upon differentiation toward T cell lineage, the iPSC-derived T cells re-expressed the same TCR as those of the parental T cells (8, 9). In addition, iPSCs are amenable to genetic modification, so it is possible to engineer the cells to have enhanced specificity and effector functions. Since iPSCs can be expanded unlimitedly, clinical-scale quantities of T cells with the desired antigen specificity can be manufactured. In this review, we provide the basic knowledge and recent advances of iPSC-derived T cell generation for clinical applications starting from the initial cell source for iPSC generation to the applications of iPSC-derived T cell products for cell-based therapy. In addition, we summarize future directions and challenges towards the final universal, off-the-shelf immunotherapeutic products.

## T Cell Biology and Applications in ACT

T cells play an essential role in the host defense mechanism against pathogens and cancers. They can be distinguished from other types of lymphocytes by the expression of TCR, which binds to the foreign antigen presented on the MHC. This interaction induces the release of cytotoxic granules and expression of Fas-ligand, which results in the target cell apoptosis (10). T cells originate from hematopoietic stem cells (HSCs), which give rise to all blood cell lineages. HSCs in the bone marrow differentiate into common myeloid progenitors (CMPs), which produce granulocyte-macrophage progenitors (GMPs) and megakaryocyte-erythroid progenitors (MEPs), or common lymphoid progenitors (CLPs), which produce lymphoid cells (12). T cell development occurs after CLPs from the bone marrow migrate into the thymus *via* the bloodstream. In the thymus, CLPs receive the Notch signal from cortical thymic epithelial cells (cTECs). During the first step of T cell development, the Notch signal stimulates CLPs to commit to double-negative (DN) cells (CD8^-^/CD4^-^) (13), which can be divided into four subpopulations (DN1 to DN4) based on the expression of CD25 and CD44 (14). From the DN1 to DN4 stages, the precursor cells undergo TCR rearrangement mediated by RAG protein to generate TCR. TCRs are randomly generated and are unique for each precursor cell. After successful TCR rearrangement, the DN4 cells express both co-receptors, CD4 and CD8 (double-positive (DP) cells). During this step, the DP cells undergo a positive selection in the cortex; the DP cells expressing TCRs that are able to bind to MHC molecules plus self-antigens on the cTEC surface with appropriate affinity will be retained (15). The outcomes of the positive selection depend on the signals from TCRs and the co-receptors (CD4 or CD8). If the DP cells have TCRs that are able to bind to MHC class II of cTECs, the DP cells will become CD4 single-positive (SP) cells by downregulating CD8 expression. On the other hand, if the DP cells have TCRs that fit the MHC class I molecule, the DP cells will downregulate the expression of CD4 and become CD8 SP cells. The DP cells that receive too low TCR signals or no TCR signals for self-antigen-MHC molecules will undergo apoptosis to prevent the generation of useless T cells (16).

Apart from positive selection, TECs also involve in negative selection, the process to eradicate the autoreactive T cells. In this process, the SP cells migrate to the medullar of the thymus where the SP cells encounter more diverse self-antigen MHC provided by medullary thymic epithelial cells (mTECs) and dendritic cells. The SP cells that bind with high affinity to the self-antigen will be eliminated from TCR repertoires by apoptosis (17). The process of negative selection generates mature T cells with a highly diverse TCR repertoire and self-tolerance to enter the bloodstream and circulate to peripheral tissues in response to pathogens (18). The newly generated T cells are considered naïve T cells at this stage because they have not been exposed to an antigen. When the naïve T cells interact with an antigen-presenting cell showing the MHC/peptide complex that can specifically bind to their TCR, T cell activation is initiated. This activation triggers the proliferation of the naïve T cell clone and differentiates the naïve T cells into the effector T cells. During this period, CD4^+^ and CD8^+^ T cells exhibit inflammatory cytokine secretion and cytotoxicity toward the transformed cells or infected cells, respectively. If the pathogen is successfully eliminated, the majority of effector T cells will die while the surviving effector T cells will be differentiated further to the memory cells. These cells are inactive and maintained for long-term immunity (19).

In 1987, Rosenberg and colleagues reported the first ACT using TILs to treat patients with metastatic malignant melanoma. TILs were expanded by *in vitro* culture in the presence of recombinant interleukin 2 (IL-2) and transfused into the patients to treat melanoma. The results demonstrated that TILs had autologous tumor-specific cytotoxicity; in addition, TILs from some patients also had limited capacity to kill allogeneic fresh tumor targets suggesting that adoptive transfer of TILs could be a potential approach for the treatment of cancer patients (20). In 1994, a larger number of patients with metastatic melanoma were treated with autologous TILs with IL-2, with or without the administration of cyclophosphamide. However, the results demonstrated that only 5 of 29 patients had complete responses (21). It was subsequently shown that lymphodepletion prior to ACT increased the complete response rate of the therapy (22, 23), and this finding led to a breakthrough in ACT against melanoma. However, TIL treatments in some types of solid cancer, such as breast cancer or cholangiocarcinoma, are not as effective as in melanomas (24), and the number of TILs is often insufficient for the treatment. To enhance the specificity of T cells and efficacy of ACT, TILs from the patients were transduced with transgenic TCR (25). These engineered TILs simultaneously react with two different antigens. Previous studies showed that the infusion of NY-ESO1 TCR-engineered T cells resulted in tumor regression in melanoma and synovial sarcoma patients (26, 27). Although genetic-engineered T cells have been developed against many antigens, their TCRs must bind to the tumor antigen presented on the HLA class I molecule to mediate the specific killing effect. This process often results in poor treatment efficacy since tumors can downregulate HLA class I molecules and co-stimulatory molecules (28, 29). To overcome this problem, CAR technology has been developed. The first generation of CAR invented in 1989 (30, 31) comprises the scFv from the antibody fused with the transmembrane domain of TCR, which contains the transduction signal, CD3ζ chain. In the second and third generations of CAR, the co-stimulatory domains derived from CD28, 4-1BB, or OX40 are added to enhance T cell activation and improve CAR T cell function against the tumors that do not express co-stimulatory molecules (32).

Although the clinical outcomes of CAR T cell therapy have been very impressive, the manufacturing costs for a single infusion of these novel therapies are very costly: $475,000 for Kymriah and $373,000 for Yescarta, making them inaccessible to most patients (33, 34). These prices do not include the hospitalization fees; therefore, the cost for the treatment needs to be reduced in order to make it economically practical and accessible to most cancer patients. Another important limitation of ACT is to find a healthy HLA-matched donor; therefore, some transplant centers focus on developing third-party T cell banks from common HLA donors (35). Other efforts have been made to generate universal allogeneic CAR T cells, which utilize healthy donor T cells for CAR and TCR engineering to increase antigen specificity and avoid graft-versus-host disease (GvHD), respectively (36–41). The treatment using these universal allogeneic CD19 CAR T cells (UCART19) demonstrated great success in two pediatric patients with acute lymphoblastic leukemia (ALL) (40). Recently, the successful results from two multicenter phase 1 studies using UCART19 in patients with relapsed and/or refractory B-ALL emphasize the potential of CAR T cells to induce complete remission in 67% of patients, even in the patients with high disease burden (42). However, there are some concerns regarding the manufacturing process; prolonged *ex vivo* culture can cause T cell exhaustion and reduced effector functions. In addition, there is also batch-to-batch variability during the manufacturing process. Therefore, clinical studies with larger cohorts are required to validate allogeneic CAR T cells (43).

## Induced Pluripotent Stem Cells as a New Cell Source for ACT

Although ACT of functional CTLs has offered a potential therapy for viral infection and cancers, the *ex vivo* expansion of autologous T cells has proved very challenging. This problem can be overcome by regenerating antigen-specific CTLs through iPSC reprogramming. Previous studies demonstrated that iPSC-derived CTLs could be expanded from 100-fold to 1,000-fold within two weeks of culture compared to 20-fold of the original T cells. These regenerated CTLs also exhibited higher telomerase activity and longer telomere length than the original T cells. Furthermore, the marker of exhausted T cells, PD-1, was not expressed, whereas the markers of central memory T cells, CCR7, CD27 and CD28, were co-expressed (9). In a more recent study, the regenerated CD8αβ CTLs were expanded up to 10,000-fold and changed their phenotype from a naïve to an effector/memory profile. In this study, 10^4^ iPSCs were used to generate 10^9^-10^10^ CD8αβ CTLs sufficient for a single transfusion (44). Apart from the regeneration of CTLs, iPSCs also provide an unlimited cell source for other T cells subsets such as regulatory T cells (Tregs) (45). Tregs play a critical role in suppressing cell-mediated immunity leading to the maintenance of immunological tolerance. Patients with autoimmune disorders have been found to have lower levels of Tregs (46). Furthermore, patients with type 1 diabetes (T1D) also have a deficient number of Tregs (47). Therefore, the generation of a large number of functional Tregs followed by ACT to autoimmune patients is required to suppress the hyperactivity of autoreactive T cells. Due to a low frequency of Treg in peripheral blood (~1-2% in humans), several attempts have been made to generate Tregs from iPSCs for use in ACT. The first proof-of-concept study showed that mouse iPSC-derived Tregs could control the development of collagen-induced arthritis in the rheumatoid arthritis mouse model (48). Similarly, the mouse iPSC-derived Tregs could migrate to the pancreas and prevent the destruction of pancreatic β-cells by autoreactive T cells in the T1D mouse model (49). Therefore, a combination of iPSC technology with adoptive immunotherapy or CAR technology may provide a large number of T cells for future clinical applications.

**Figure 1:**
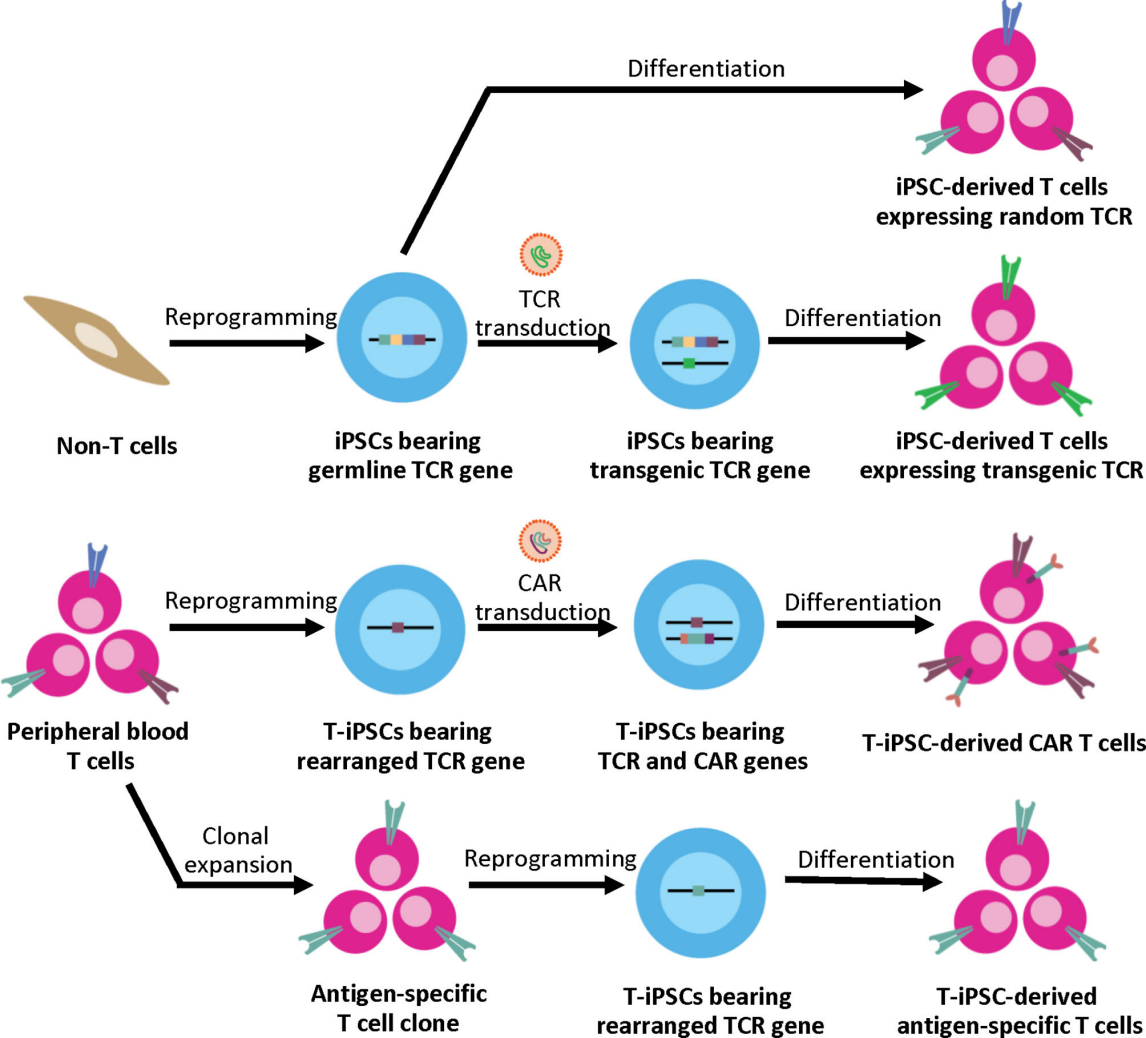
Generation of iPSC-derived T cells from different somatic cell sources. Non-T cell sources contain germline TCR gene, upon T cell differentiation, the iPSC-derived T cells express random TCR. These T cells can be used for studying normal T cell development and disease modeling. For applications in ACT, the exogenous TCR can be introduced to the iPSCs. Upon T cell differentiation, the transgenic TCR generates the CD3 signal, which then leads to allelic exclusion and inhibition of endogenous TCR rearrangement; therefore, the iPSC-derived T cells express the transgenic TCR to target specific antigens. Alternatively, peripheral blood T cells can serve as a cell source for iPSC generation. T cell has the rearranged TCR gene, which is retained throughout the reprogramming and differentiation process. For applications in ACT, T-iPSCs can be engineered with CAR to enhance tumor specificity, or the antigen-specific T cell clone can be used for reprogramming to generate the antigen-specific T cells.

Unlike other differentiated cell types, the generation of functional CTLs with a specific TCR from iPSCs depends significantly on the original somatic cell sources (**Figure 1**). When using non-T cell sources such as fibroblasts or keratinocytes as a somatic cell source, the derived iPSC clones bear the germline TCR gene. After T cell differentiation *in vitro*, the iPSC-derived T lymphocytes are generated with unpredictably rearranged TCR. This process recapitulates normal T cell development where sequential expression of CD7, cytoplasmic CD3, and surface CD3 was observed followed by TCR gene rearrangement of the γδ and αβ loci, respectively (50). However, without autologous TECs, positive and negative selection may not occur. Therefore, these iPSCs can only be used for studying normal T cell development and disease modeling; they are not suitable for clinical use due to the concern about autoreactive T cells. Apart from studying normal T cell development, disease-specific iPSCs can be generated from somatic cells (non-T cells) of patients with inherited diseases affecting the immune system such as X-linked Severe Combined Immunodeficiency (SCID-X1) with the Interleukin-2 receptor gamma chain (*IL-2Rγ*) mutation (51) or recombination-activating gene 1 (*RAG1*) mutations (52) to study abnormal T cell development in these disease models. Genetic correction in these disease-specific iPSCs using genome editing technologies such as TALENs or CRISPR/Cas9 systems with a subsequent *in vitro* differentiation also offers great potentials for future autologous therapy (51).

In 2011, Seki et al. developed the method for iPSC generation from mature human peripheral blood T cells using a Sendai viral vector to avoid transgene insertion (53). This method could generate iPSCs from a small amount (approximately 1 ml) of human peripheral blood samples (54). However, their method used fetal bovine serum (FBS) and mouse embryonic fibroblasts (MEF) as feeder cells, which result in contamination of xenogeneic antigens and zoonotic pathogens. In 2014, the generation of human iPSCs from peripheral blood T cells in a defined culture system was achieved using Sendai viral transduction and various combinations of chemically defined culture medium and coating matrices. For example, the combination of mTeSR1 medium and Matrigel resulted in the highest reprogramming efficiency (0.005%) (55). Overall, the reprogramming efficiencies under the feeder-free system are generally lower than those using the feeder cells. Even though the reprogramming efficiency using blood cells is lower than fibroblasts, blood cells are preferable because the isolation is minimally-invasive and easy to perform.

On the other hand, generation of iPSCs from T cells results in the pre-rearranged TCR gene in the iPSC clones. The rearranged TCR can eliminate the risk of autoreactive TCR since the T cells undergo positive and negative selection in the thymus. However, the specificity of TCR is unknown. In 2013, Themeli et al. reported the generation of CD19 CAR-engineered T-iPSCs that can efficiently be differentiated into CAR T cells against CD19^+^ malignant B cells *in vitro*. These T-iPSC-derived CAR T cells displayed therapeutic activity by potently inhibiting tumor growth in a mouse model (56). Similarly, Minagawa et al. demonstrated that when the monocyte-derived iPSCs were transduced with a transgenic antigen-specific TCR, these cells exhibited a monoclonal expression of the transduced TCR after T cell differentiation *in vitro*. The iPSC-derived transgenic TCR T cells could also delay tumor progression in xenograft cancer models (57). These two studies showed that even though the iPSCs have no antigen-specific TCR, the specificity of iPSC-derived T cells can be achieved by transduction of CAR or transgenic TCR to generate therapeutic T cells for cancer immunotherapy.

After the concept of T cell production utilizing PSCs has been proposed, Watarai et al. utilized the nuclear transfer technique to reprogram NKT cells. The nuclear transfer ESCs bearing rearranged invariant Vα14-Jα18 TCRα gene were established from the mouse NKT cells (58). This study has proved that the rearranged TCR gene was retained throughout the reprogramming and differentiation process. Advances in the iPSC technology in 2006 led to reprogramming of CD8^+^ T cells specific to MART1^+^ melanoma using Sendai viral vectors carrying OSKM factors and SV40 large T antigen at MOI 30. Analysis of TCRα chain mRNA in the CD8^+^ T cells generated from these iPSCs confirmed that the iPSC-derived CD8^+^ T cells expressed the same TCRα chain gene as the parental MART1-specific T cells (8). Similarly, Nishimura et al. reported successful reprogramming of antigen-specific T cells into iPSCs. First, the transduction was performed using six retroviral vectors encoding OCT3/4, SOX2, KLF4, c-MYC, NANOG, and LIN28A; however, no iPSC-like colonies were observed. In the second attempt, the reprogramming was performed using the Sendai viral (SeV) vector system consisting of two Sendai viral vectors. The first vector encodes OSKM factors and the microRNA-302, while the second vector encodes the SV40 large T (LT) antigen. The iPSC-like colonies appeared on the mouse embryonic fibroblast (MEF) feeder cells within 40 days after transduction (9). The same approach enables reprogramming of several T cell clones specific for Nef antigen in HIV, pp65 antigen in cytomegalovirus (CMV), glutamic acid decarboxylase (GAD) antigen in type 1 diabetes, and alpha-Galactosylceramide (α-GalCer). Importantly, the iPSCs and the parental T cells had identical antigen-recognition sites (CDR3 sequence) on the *TCRA* and *TCRB* genes (9). Recently, the SeV vectors encoding five factors (OSKM + SV40 LT antigen) were used for reprogramming various types of antigen-specific T cells and NKT cells, including WT1-specific T cells, LMP2-specific T cells (44), GPC3-specific T cells (57), b3a2-specific T cells (59) and Vα24^+^ invariant natural killer T cells (60, 61).

In contrast to peripheral blood T cells, antigen-specific T cells are mainly effector memory T cells or central memory T cells, which are in the latest stage of development. Effector memory T cells or central memory T cells are prone to apoptosis when stimulated due to their short telomere length (62). Therefore, reprogramming of antigen-specific T cells is very technically challenging. Previous studies showed that the process requires supplementation of the OSKM factors with additional factors such as hTERT and SV40 LT antigen, which have potent anti-apoptotic activity (63), and the use of MEF feeder cells (8, 9). It is worth noting that the hTERT and SV40 large T antigen are known oncogenes; upon insertion into the genome, these factors may cause tumorigenesis. Even though it is not a concern when using the SeV vector system because the viral RNA is diluted and removed from the cells after reprogramming, the SV40 large T antigen might increase double-stranded break (DSB)-associated mutations. Thus, other pluripotency-associated genes, such as NANOG and LIN28, were used instead of SV40 LT antigen in combination with OSKM factors for T cell reprogramming (64). This system called 6-factor (OSKM + NL) offers advantages over the conventional system (OSKM + SV40 LT) by eliminating the oncogenes and is therefore preferable for applications in ACT. In addition, T-iPSCs reprogrammed by a 6-factor system were able to efficiently differentiate into antigen-specific T cells with strong cytotoxicity against cervical cancer. There is no significant difference in cytotoxicity from that of the conventional T-iPSCs (64). Although this 6-factor system successfully generated iPSCs from the antigen-specific T cells, there are two main issues associated with using antigen-specific T cell-derived iPSCs for clinical translation, including clonal variability, which affects T cell differentiation potential (65), and alloreactivity (66). The study demonstrated that approximately 50% of antigen-specific T cell-derived iPSC clones exhibited great T cell differentiation potential (66). There is also a possibility that T cell alloreactivity will occur at 10% even in the case of HLA-matched patients (67, 68). Therefore, to develop an off-the-shelf product from T-iPSCs for use in an allogeneic setting, it is necessary to establish multiple clones of antigen-specific T cell-derived iPSCs and screen for the best clones and other spare clones in case of alloreactivity. It was estimated that eight initial iPSC clones are sufficient to create two powerful T-iPSC clones (66). The generation and screening of eight iPSC clones are time-consuming and expensive, especially from antigen-specific T cell sources. An alternative approach such as introducing TCR or CAR into T-iPSCs would be more practical for developing off-the-shelf ACT.

## Generation of T Cells From Pluripotent Stem Cells

**Figure 2:**
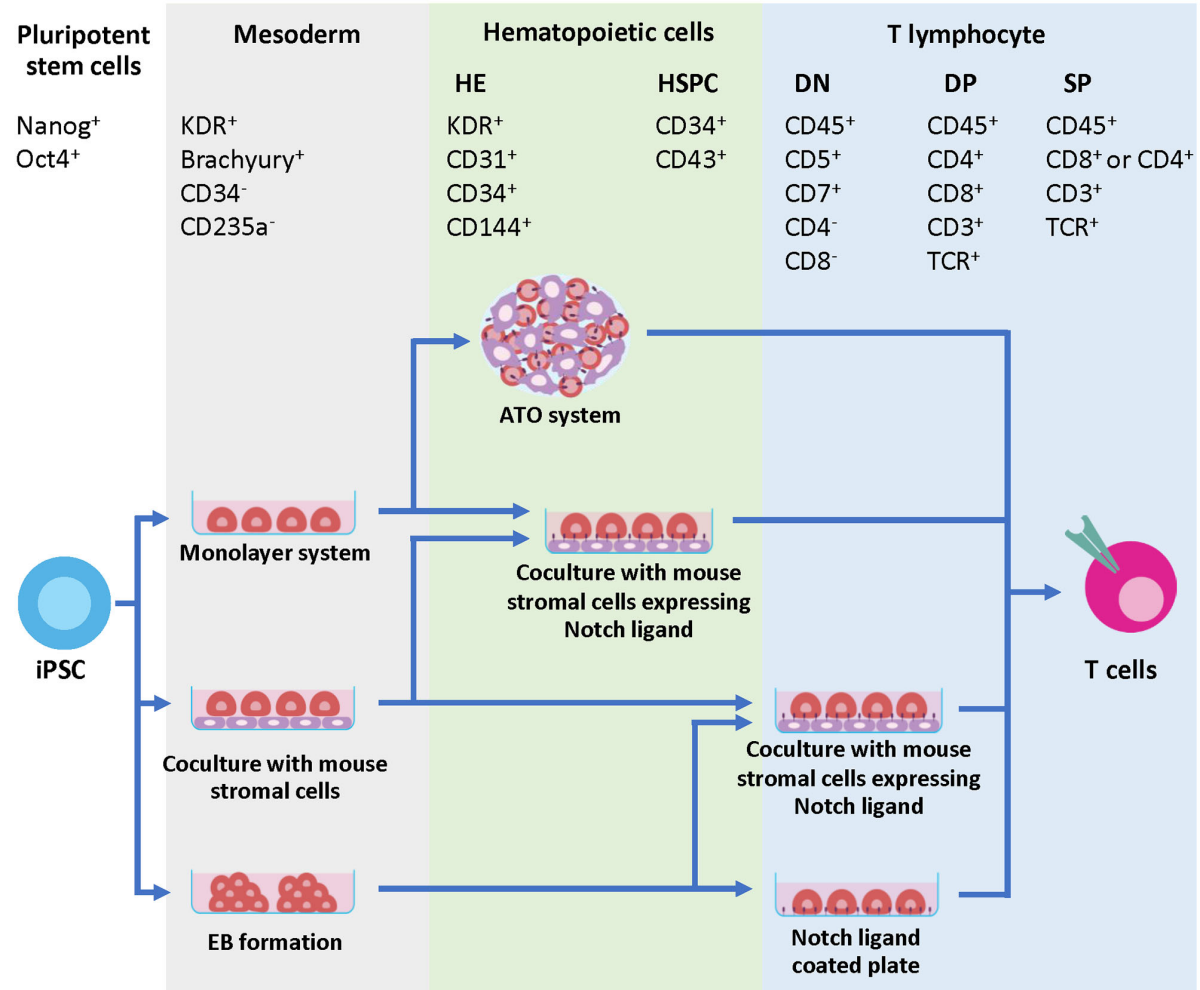
Developmental markers during T cell differentiation and strategies to generate iPSC-derived T cells. The initial step of hematopoietic differentiation can be achieved by various protocols, including feeder-free protocols such as monolayer system, co-culture with mouse stromal cells and EB formation. During this step, the mesodermal (ME) cells expressing Brachyury and KDR are generated. The ME cells are committed further to HE, which express KDR, CD31, CD34 and CD144. During EHT process, CD43^+^ HSPC emerges from the HE layers. Specification of T cell lineage requires Notch signaling, which can be provided through co-culture with mouse stromal cells such as OP9-DL1 or OP9-DL4. Co-culture of iPSC-derived multipotent HSPCs with these cells in 2D or 3D system efficiently generates mature T cells with phenotypes CD8^+^ CD4^-^ TCR^+^ and CD3^+^. Alternatively, the Notch signals can be provided through a coating matrix mixture of retronectin and recombinant DL4 protein.

Generation of T cells from PSCs requires two essential stages. First, PSCs need appropriate signals from microenvironments to be committed toward hematopoietic stem cells (HSCs), followed by the Notch signaling for T cell lineage commitment (69). During the first step toward HSCs, PSCs must be differentiated into the definitive mesoderm (ME) and hemogenic endothelium (HE), which then undergoes the process known as an endothelial-to-hematopoietic transition (EHT). During EHT, the HE is rounded up and releases the floating cells with hematopoietic stem/progenitor cell (HSPC) markers, CD34 and CD43, into the medium (70, 71). Two waves of hematopoiesis occur in human embryo development, primitive and definitive. Definitive hematopoiesis can give rise to HSPCs with the potential to develop into T cells (72). The previous study demonstrated that there are no true markers to distinguish between the primitive and definitive HSPCs in the CD34^+^ CD43^+^ populations. Therefore, identification of ME by using the phenotypes KDR^+^ and CD235a^-^ is essential (73). After HSC induction, the differentiation process must recapitulate normal T cell development in the thymus where sequential expression of CD7, cytoplasmic CD3, and surface CD3 was observed, followed by TCR gene rearrangement of the γδ and αβ loci, respectively. This section focuses on various approaches that have been used to mimic the microenvironment in the thymus to induce mature T cell differentiation *in vitro* (**Figure 2** and **Table 1**).

**Table 1:** Generation of T cells from human iPSCs.

Cell source of iPSCs	Regenerated T cells	T cell differentiation	Functional test	Ref
**Non-T cells**				
- **Keratinocytes**	Randomly rearranged TCR T cells	Co-culture with OP9-DL4 cells	*In vitro* TCR activation assay	(74)
- **Myeloid cells**	WT1-TCR transduced T cells	Co-culture with OP9-DL1 cells	*In vitro* and *in vivo* specific killing assay	(57)
		Culture onto DL4-coated plate	*In vitro* and *in vivo* specific killing assay	(75)
- **Monocytes**	WT1-TCR transduced T cells	Co-culture with OP9-DL1 cells	*In vitro* and *in vivo* specific killing assay	(76, 77)
- **Fibroblasts**	T cells	Co-culture with OP9-DL1 cells	*In vitro* TCR activation assay	(78)
		Co-culture with MS5-DL4 cells in ATO	N/A	(79)
**T cells**				
- **PHA-activated lymphocytes**	CD19-CAR transduced T cells	Co-culture with OP9-DL1 cells	*In vitro* and *in vivo* specific killing assay	(56)
- **Purified CD3^+^ T cells**	T cells	Co-culture with OP9-DL1 cells	*In vitro* TCR activation assay	(80)
- **MART-1 specific CTL clone**	MART-1-specific T cells	Co-culture with OP9-DL1 cells	*In vitro* TCR activation assay	(8)
- **Sorted MART-1-tetramer^+^ T cells**	MART-1-specific T cells	Co-culture with OP9-DL1 cells	*In vitro* specific killing assay	(66)
- **Nef-specific CTL clone**	Nef-specific T cells	Co-culture with OP9-DL1 cells	*In vitro* specific killing assay	(9, 81)
		Culture onto DL4-coated plate	*In vitro* specific killing assay	(75)
	iC9-transduced Nef specific T cells	Co-culture with C3H10T1/2-DL1 cells	*In vitro* specific killing assay	(82)
- **GAG-specific CTL clone**	GAG-specific T cells	Co-culture with OP9-DL1 cells	*In vitro* specific killing assay	(81)
		Culture onto DL4-coated plate	*In vitro* specific killing assay	(75)
- **GPC3-specific CTL clone**	RAG2 KO GPC3-specific T cells	Co-culture with OP9-DL1 cells	*In vitro* and *in vivo* specific killing assay	(57)
	GPC3-specific T cells	Culture onto DL4-coated plate	*In vitro* specific killing assay	(75)
- **LMP1-specific CTL clone**	LMP1-specific T cells	Co-culture with C3H10T1/2-DL1/4 cells	*In vitro* specific killing assay	(83)
- **LMP2-specific CTL clone**	LMP2-specific T cells	Co-culture with OP9-DL1 cells	*In vitro* specific killing assay	(44)
		Co-culture with C3H10T1/2-DL1/4 cells	*In vitro* and *in vivo* specific killing assay	(83)
	iC9-transduced LMP2-specific T cells	Co-culture with C3H10T1/2-DL1 cells	*In vitro* and *in vivo* specific killing assay	(82)
- **WT1-specific CTL clone**	WT1-specific T cells	Co-culture with OP9-DL1 cells	*In vitro* and *in vivo* specific killing assay	(44, 76, 77)
- **Sorted HPV16-E6 -tetramer^+^ T cells**	HPV16-E6-specific T cells	Co-culture with C3H10T1/2-DL1/4 cells	*In vitro* and *in vivo* specific killing assay	(64)
- **Sorted HPV16-E7 -tetramer^+^ T cells**	HPV16-E7-specific T cells	Co-culture with C3H10T1/2-DL1/4 cells	*In vitro* specific killing assay	(64)
- **b3a2-specific Th1 clone**	CD4-transduced b3a2-specific T cells	Co-culture with OP9-DL1 cells	Priming CTLs to increase specific killing *in vitro* and *in vivo*	(59)
- **Expanded TILs from colorectal cancer specimens**	Multiclonal colorectal cancer-specific T cells	Culture onto DL4-coated plate	*In vitro* and *in vivo* specific killing assay	(84)

ATO, artificial thymic organoid; b3a2, junction region of BCR-ABL p210; CAR, chimeric antigen receptor; CTL, cytotoxic T lymphocyte; DL1, delta-like 1; DL4, delta-like 4; GAG, group-specific antigen; GPC3, glypican-3; HPV16-E6, human papillomavirus type 16 early protein 6; HPV16-E7, human papillomavirus type 16 early protein 7; iC9, inducible caspase-9; KO, knockout; LMP1, latent membrane protein 1; LMP2, latent membrane protein 2; MART-1, melanoma antigen recognized by T cells 1; Nef, negative regulatory factor; PB, peripheral blood; PHA, phytohaemagglutinin; RAG2, recombination activating gene 2; TCR, T cell receptor; Th1, T helper type 1; TIL, tumor-infiltrating lymphocytes; WT1, Wilms’ tumor 1.

### Co-Culture System Using Stromal Cells

A simple and well-known method to induce T cell commitment *in vitro* is the co-culture system with mouse stromal cells, OP9, as supporting cells for T cell differentiation. The OP9 cell line can be derived from the mouse bone marrow with a defect in macrophage colony-stimulating factor (MCSF) production (85). The OP9 cells can be expanded *in vitro* for a long time and selectively facilitate HSPC differentiation and lymphoid development (86). In 2002, Schmitt et al. developed a monolayer co-culture system for *in vitro* T cell differentiation using the OP9 cell line overexpressing Delta-like 1 (OP9-DL1), a human homolog of the Notch ligand. Co-culture of mouse HSCs with the OP9-DL1 cells induced CD4^+^ CD8^+^ double-positive (DP) T cells and CD8^+^ SP T cells (87). In 2005, La Motte-Mohs et al. published the first report of the generation of human T cells from CD34^+^ HSPCs using the OP9-DL1 co-culture system (88). A similar co-culture system has been used to generate T cells *in vitro* using the MS5 and C3H/10T1/2 stromal cell lines expressing DL1. Similar to OP9-DL1, MS5 and C3H/10T1/2 stromal cells overexpressing DL1 support the differentiation of umbilical cord blood CD34^+^ HSPCs to CD7^+^ DN cells after 3-4 weeks of co-culture (82, 89). Apart from DL1, Delta-like 4 (DL4) is also known as a ligand for Notch-1 receptor (90). The *in vitro* study showed that DL4 overexpression in stromal cells could support T cell development in a similar manner to DL1 (69, 91). Although there was no significant difference between the yield of T cell differentiation when co-culturing with OP9-DL1 and OP9-DL4, DL4 provided better results at physiological expression levels (92). Further study indicated that DL4 provided a 10-fold greater Notch receptor binding affinity than DL1 (93). As a result, some studies used OP9-DL4 as a feeder cell for T cell differentiation from pluripotent stem cells (50, 52, 72).

The first successful generation of T cells from iPSCs was reported in 2009 by Lei et al., where mouse iPSCs co-cultured with the OP9-DL1 cells in the presence of Flt3L and IL-7 could be differentiated into the TCRβ^+^ CD8^+^ SP T cells. These cells produced IL-2 and IFN-γ after activation with anti-CD3 antibody, indicating that they are functional T cells. In addition, the iPSC-derived T cells restored the T cell pool in Rag1^-/-^ mice after infusion (94). In contrast to mouse iPSCs, a single-step co-culture system with the OP9-DL1 cells has not been achieved in human iPSCs. To generate T cells, human iPSCs were differentiated to CD34^+^ HSPCs *via* three methods, embryoid body (EB) formation (56, 72), monolayer system (80, 95), and direct co-culture with the OP9 (96) or C3H10T1/2 cells (9). The CD34^+^ cells were then transferred onto the OP9-DL1 cells in the presence of Flt3L and IL-7 to further differentiate into pro-T cells, which later required TCR signal to become mature T cells (8, 9, 97). The mouse iPSC-derived pro-T cells can acquire TCR signals from the MHC molecule on the OP9 cells (94, 98). In contrast, the human iPSC-derived DP T cells cannot recognize the mouse MHC molecule on the OP9 cells, so they cannot obtain the TCR signal from co-culturing with the OP9-DL1 cells. Therefore, activation of human pro-T cells using anti-CD3 antibody is required to generate mature T cells (8, 9, 44).

Although TCRαβ^+^ CD8^+^ T cells can be derived from human iPSCs, previous studies showed that human iPSCs could generate only T cells expressing CD8α subunit (CD8αα T cells) and high levels of innate T cell-related markers (such as CD56) (8, 44, 56, 82). The CD8αα T cells differentiated from human iPSCs were different from the effector T cells in peripheral blood, which are CD8αβ T cells. More importantly, the regenerated CD8αα T cells from iPSCs showed a gene expression pattern similar to those of the innate T cells and exhibited a non-specific killing effect (44, 56).

Recently, Maeda et al. reported a novel method to generate the CD8αβ T cells from human iPSCs. During the differentiation step, the CD4^+^ CD8^+^ DP cells were sorted and activated using anti-CD3 antibody to generate CD8αβ T cells similar to the effector T cells from peripheral blood (44). DNA sequencing revealed that the TCR gene of the iPSC-derived T cells and the parental T cell clone were completely identical, suggesting that antigen specificity of the parental T cells was retained in the iPSC-derived T cells (8, 9, 44). Thus, *in vitro* cytotoxicity of regenerated T cells was comparable to the parental antigen-specific T cells (44). Moreover, the regenerated T cells had a rejuvenated phenotype. The iPSC-derived T cells established from an HIV-1-specific CTL clone could be expanded from 100-fold to 1000-fold within two weeks, whereas the parental T cells could be expanded up to 20-fold. The regenerated CTLs also had a 1.5-fold longer telomere length than parental CTLs (9). Finally, the treatment with the iPSC-derived CD8αβ T cells markedly delayed tumor growth in the mouse model (44, 57, 82). Worth noting that there is no report of the successful generation of CD4^+^ helper T cells from iPSCs even though T-iPSC was derived from the CD4^+^ T cell clones (59). Antigen-specific CD4^+^ T helper cells are essential in controlling immune reactions. These cells can amplify anti-tumor immunity by inducing the activation of tumor antigen-specific CTLs. Therefore, the absence of CD4^+^ T cells in the iPSC-derived T cell population may lead to insufficient control of tumor growth in patients.

### Artificial Thymic Organoid

The three-dimensional (3D) structure of primary thymic stromal cells has been shown to promote positive selection and TCR rearrangement of human T cells *in vitro* (99). In 2017, Seet et al. developed a new method called artificial thymic organoids (ATO) system that combines the 3D organoid culture elements and the expandability of the stromal cell line. The ATO system requires a serum-free medium and the MS5 mouse stromal line expressing human DL1 or DL4 (MS5-DL1 or DL4 cells), which formed small 3D aggregates with human HSPCs by centrifugation. The 3D aggregates were plated onto micropore filters and cultured for six weeks. This ATO system fully recapitulated the T cell development, especially during the TCR rearrangement. At week 6 in ATOs, up to 20% of total cells expressed TCRαβ and CD3, indicating that the cells reached the SP stage without the requirement of anti-CD3 antibody. In addition, CD8 SP T cells and CD4 SP cells isolated from ATOs produced IFN-γ and IL-2 in response to PMA and ionomycin activation (100).

The ATO system was also applied to generate mature T cells from ESCs and iPSC (79). Firstly, the ESCs or iPSCs were induced to mesodermal lineage using BMP4, VEGF and bFGF for three days in the monolayer culture system. The cells were then dissociated into single cells and centrifuged with the MS5-DL4 cells to form aggregates, which were cultured in the hematopoietic induction medium for two weeks followed by the T cell induction medium for 50 days. This approach generated CD8 and CD4 SP T cells, which produced IFN-γ in response to phorbol 12-myristate 13-acetate (PMA) stimulation. Deep sequencing results revealed that the TCRα and β chain rearrangement occurred during the T cell differentiation in the ATO system. Moreover, when using the NY-ESO-1-specific TCR engineered H1 ESC line in the ATO system, nearly 100% of the generated T cells expressed NY-ESO-1-specific TCR. Transduction of NY-ESO-1-specific TCR also inhibited the rearrangement of the endogenous TCRαβ due to allelic exclusion of the TCR gene. Following 14 days of expansion, the ESC-derived TCR-engineered T cells expanded approximately 100-fold and displayed specific cytotoxicity against the NY-ESO-1 expressing target cells *in vitro* and in immunodeficient mice. Interestingly, the studies demonstrated that the ATO system could support the robust differentiation of CD4^+^ T cells (79, 101). However, the function and potential of CD4^+^ helper T cells generated from this method have not been clearly investigated.

### Feeder-Free Differentiation System

Despite the success in generating T cells, the use of mouse cells as supportive feeders is not compatible with the development of clinical-grade products due to contamination of xenogeneic antigens. Although there have been many attempts to develop human feeder cells to replace the mouse cell lines, the results were unsatisfactory. Human fibroblasts or keratinocytes engineered to express DL4 were insufficient to promote the differentiation of human HSPCs to DN or DP T cells (102, 103). The first attempt to differentiate mouse HSPCs toward T cells under the feeder-free system was performed using the recombinant Notch ligand DL1 fused with Fc domain of human IgG (DL1-Fc)-coated culture dish. This system enabled the generation of the DP T cells that could reconstitute mature T cells in the NOD/SCID mouse model (104). A similar approach to differentiate mouse HSPCs applied the DL4-Fc protein-immobilized culture dish in the medium supplemented with SCF, Flt3L and IL-7. This system efficiently promoted the DP T cell development (105). For a scalable T cell differentiation system, Taqvi et al. immobilized the DL4 protein on microbeads to support T cell development from bone marrow-derived HSPCs. The results showed that the DL4-conjugated bead system was sufficient to induce T cell commitment; however, most differentiated cells were committed to the B cell lineage leading to inefficient T cell generation (106).

Another group developed a novel feeder-free method combining the recombinant VCAM-1 with DL4 proteins. This system synergistically increased the robustness of T cell commitment from cord blood-derived HSPCs in a xenogeneic-free differentiation medium. After two weeks of differentiation, the differentiated cells were arrested at the DP stage with the phenotype of CD34^-^ CD7^+^ CD5^+^ cells. The purified CD7^+^ cells were further differentiated *in vivo* by intrahepatically injecting into neonatal immunocompromised mice. After 10–12 weeks post-engraftment, functional mature T cells were detected and circulated in the peripheral blood of the immunodeficient mice (107). Recently, Iriguchi et al. reported the success of using a feeder-free system to generate iPSC-derived mature T cells. The iPSC-derived CD235a^−^/CD14^−^/CD34^+^/CD43^+^ cells were purified and differentiated into the functional antigen-specific T cell lineage under a feeder-free system using immobilized DL4 protein and retronectin. During the differentiation, 3 × 10^5^ iPSCs could give rise to 6.2 × 10^8^ T cells. Importantly, these iPSC-derived T cells demonstrated the anti-tumor function in both *in vitro* and *in vivo* xenograft models (75). Similarly, Ito et al. demonstrated that this feeder-free protocol could be applied for the generation of tumor-specific T cells from TIL-derived iPSCs. The result showed that the regenerated T cells retained the T cell function and tumor-specific killing. Moreover, there was no additional rearrangement at either the TCRα or TCRβ chains of the T cells generated by this feeder-free protocol (84). However, these two studies still used bovine serum albumin in the medium to obtain a large number of mature T cells; therefore, the development of a complete xenogeneic-free condition for clinical translation of iPSC-derived T cells is still very challenging.

## Advances of iPSC-Derived CAR T Cells for Off-the-Shelf ACT

The advent of genetic engineering has created the so-called next-generation stem cell-based therapies with enhanced therapeutic efficiencies (108). The most promising therapeutic application in oncology to date has been CAR technology. To date, there are four CD19 CAR T cell products approved by the FDA for the treatment of relapsed or refractory large B cell lymphoma (2), and more than 900 ongoing clinical trials targeting different types of cancers (ClinicalTrials.gov). While CAR T cell therapy holds impressive clinical outcomes, many challenges hinder its applications, including insufficient autologous T cells due to lymphopenia in patients and a high production cost. Human iPSCs have become an attractive cell source for the generation of CAR T cells regarding their self-renewal capacity. In 2013, Themeli et al. reported the first proof-of-concept study showing that the CD19 CAR-engineered iPSCs could be used as a starting cell source for generating the functional CD19 CAR T cells with anti-cancer capability in a xenograft model (56). To broaden the applicability of CAR T cell therapy, many attempts have been made to generate allogeneic CAR T cells devoid of TCR to eliminate the risk of GvHD. These strategies employ genome editing technologies such as zinc finger nucleases (37), TALENs (40) or CRISPR/Cas9 (36) to disrupt TCR expression in primary T cells from healthy donors and introduce CAR specific to cancer antigens. Using CRISPR/Cas9 technology, Sadelain and colleagues generated the engineered T cells with CD19 CAR gene knockin at the TCR α constant (TRAC) locus. The engineered T cells lack the endogenous TCR expression and simultaneously express CD19 CAR under the control of its transcriptional regulatory elements. These engineered TRAC-encoded CD19 CAR T cells exhibited increased anti-tumor activities in the leukemic mouse model regarding the responses and prolonged medium survival compared to the conventional, randomly integrated CD19 CAR T cells. This study emphasized the importance of transcriptional regulation of CAR expression; the use of endogenous regulatory elements resulted in a better-defined T cell product with minimized TCR-induced autoimmunity and alloreactivity as well as delayed exhaustion (36). Although the absence of TCR expression can lower the risk of GvHD, CD3 signaling from CAR can alter the T cell lineage commitment. The presence of all three CD3ζ immunoreceptor tyrosine-based activation motifs (ITAMs) has been shown to compromise the therapeutic potency of CAR T cells. Therefore, the team modified the second and third CD3ζ ITAMs of CAR to be non-functional (1XX) and generated CD19 1XX CAR T cells. These engineered CAR T cells have calibrated ITAM activity with similar strength of CD3 signaling from TCR, thereby exhibiting increased persistence and better therapeutic efficacy in the well-established pre-B acute lymphoblastic leukemia (B-ALL) mouse model compared to the CAR T cells with all three CD3ζ ITAMs or other types of mutants (109).

Despite excellent results obtained in primary CAR T cells, multiplex genome engineering, quality control, and validation are technically challenging. One way to address this issue is to harness the unique characteristics of iPSCs, which are amenable to genetic manipulation and clonal validation. Fate Therapeutics has combined the iPSC technology with CAR to generate the iPSC-derived TCR-less CD19 1XX CAR T cell product to treat B-ALL. Upon T cell differentiation, the iPSCs harboring TRAC-CD19 1XX CAR could give rise to the highest CD4^+^ CD8^+^ DP population compared to other types of iPSC-derived CAR T cells. Importantly, the CD4^+^ CD8^+^ DP cells could be efficiently differentiated into CD8αβ SP CAR T cells (110). This novel platform, so-called “the first-of-kind off-the-shelf hiPSC-derived CAR19 T cell product FT819” was manufactured under the current Good Manufacturing Practice (cGMP) compliance and applied in the pre-clinical study. The *in vivo* leukemia xenograft mouse studies also showed that FT819 could control tumor burden and prolong survival rate similar to those of the CD19 CAR T cells (111, 112). In addition, the mixed lymphocyte reactions performed with HLA-mismatched peripheral blood mononuclear cells (PBMCs) confirmed the lack of alloreactivity, thereby eliminating the risk of GvHD (113). Recently, Phase I multicenter trial of FT819 has been initiated in up to 300 patients with relapsed/refractory B cell malignancies. Various FT819 dose levels ranging from 30 to 900 million cells will be tested to find the recommended Phase II dose. Three treatment regimens for each type of cancer will be included: Regimen A, FT819 will be given as a single dose; Regimen B, FT819 will be given as a single dose combining with IL-2; and Regimen C, FT819 will be given at three fractionated doses (114).

Besides the risk of GvHD, graft rejection by the recipient’s immune cells is another concern. Several groups have generated universal or hypoimmunogenic iPSC lines by eliminating HLA class Ia (HLA-A, -B, and -C) and class II molecules to avoid immune rejection by CD8 T cells and CD4 T cells, respectively, and introducing HLA class Ib (HLA-G or HLA-E) or immune checkpoint molecules (PD-L1 or CD47) to prevent NK cell-mediated lysis or phagocytosis by macrophages (115–122). To date, the main challenge for translating these approaches is how to avoid NK cell-mediated lysis. This can be achieved by suppressing the activating signals or promoting the inhibitory signals. However, there are diverse activating and inhibitory receptors expressed on NK cells of each individual; thus, targeting multiple receptors is necessary to completely prevent the NK cell attacks (123, 124). Previous studies showed that expression of HLA-E in the HLA-null iPSC-derived CD45^+^ cells (116) and iPSC-derived retinal pigment epithelial cells (125) could inhibit NK cell-mediated lysis through the interaction with CD94/NKG2A receptors. However, it was shown that approximately 50% of NK cells express NKG2A receptor (126); therefore, HLA-E expressing cells may still be a target for NKG2A^-^ NK cells (122). More recently, Wang et al. took a step forward by knocking out poliovirus receptor (PVR) or CD155, a ligand for NK cell-activating receptor DNAM-1, in the HLA-E-transduced, HLA-I- and HLA-II-null iPSCs. Upon differentiation toward cytotoxic T cells, the engineered cells could reduce the activation of DNAM-1^+^ NK cells, consisting of both NKG2A^+^ and NKG2A^-^ populations, and persisted longer than the HLA-intact iPSC-derived T cells *in vitro* and *in vivo* in the presence of allogeneic immunity (119). Therefore, engineering multiple inhibitory/activating signals could lead to a more effective escape from NK cells making the iPSC-derived T cells applicable to a larger number of patients.

Apart from the modification of TCR and/or HLA genes and the introduction of CAR for the generation of universal iPSC-derived CAR T cells, there are attempts to engineer the iPSCs with other molecules to expand the potential of adoptive iPSC-derived CAR T cell therapy. One feature is the expression of a high-affinity, non-cleavable form of antibody receptor CD16 (hnCD16), which allows the scientists to adjust the specificity of the T cell killing through antibody-dependent cellular-cytotoxicity (ADCC) by adding a monoclonal antibody. For example, the iPSC-derived CD19 CAR-hnCD16 T cells could efficiently recognize and kill both CD19^+^ CD20^+^ and CD19^-^ CD20^+^ tumor cells when combined with anti-CD20 monoclonal antibody (Rituxan) (113). Therefore, this strategy could be applied to target multiple cancer antigens. Another approach to increase the persistence and therapeutic efficacy of iPSC-derived CAR T cells is to engineer a signaling-fusion complex such as IL-7 receptor fusion (IL-7RF), which is a fusion protein of IL-7 receptor and its ligand; therefore, IL-7RF can generate IL-7 signal by itself without exogenous IL-7 support. The addition of IL-7RF led to higher anti-tumor activity compared to the control group in both the *in vitro* and *in vivo* studies (127).

**Figure 3:**
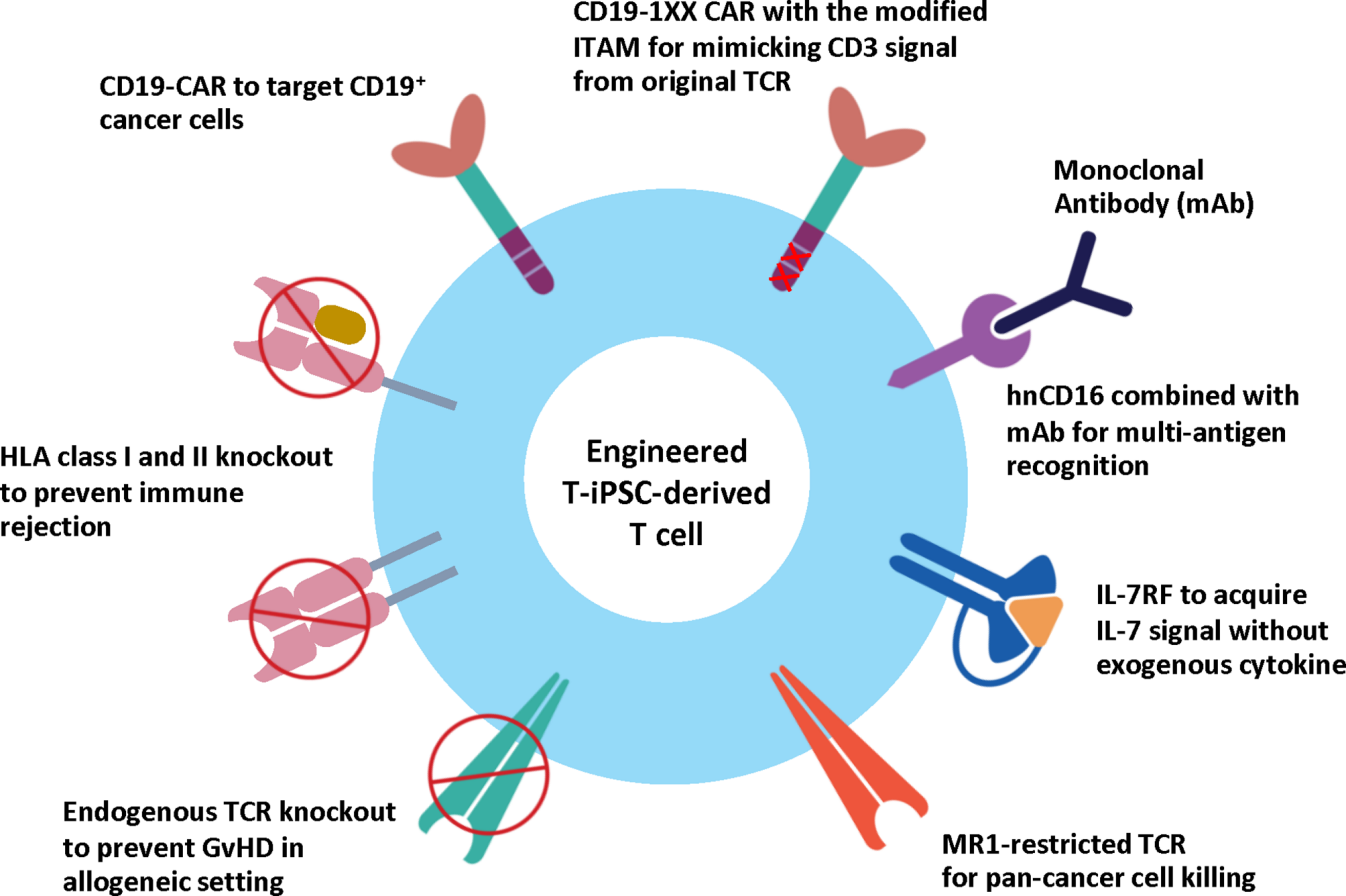
Engineered T-iPSC-derived T cells for next-generation ACT. Genome editing technologies can be used to eliminate the endogenous TCR to reduce the risk of graft-versus-host-disease (GvHD) or HLA molecules to reduce the risk of immune rejection for allogeneic use, or to introduce CAR to specifically target cancer cells. However, the conventional CAR with three ITAM motifs generates higher CD3 signals than endogenous TCR and results in altered T cell differentiation of iPSCs. CD19-1XX CAR construct is the novel CAR construct with mutated second and third ITAM motifs to reduce the CD3 signal. Apart from CAR, the iPSC-derived T cells can be modified to express MR1-restricted TCR to target a wide range of cancer cells. Other strategies to enhance cytotoxic activity and persistence include the expression of hnCD16 and IL-7 RF.

In 2020, a novel TCR (MC.7.G5) was discovered using a genome-wide CRISPR-Cas9 screening. This TCR exhibits a pan-cancer cell recognition potential *via* the invariant monomorphic MHC class I-related protein MR1 molecule. T cells expressing the MR1-restricted TCR (MR1-TCR) could kill a broad range of cancer cells independently of classical MHC molecules. Importantly, these MR1-TCR T cells are inert when being co-cultured with healthy cells from various tissues (128). The discovery of the MR1-TCR offers therapeutic opportunities for many cancers in all individuals. Recently, Nguyen et al. demonstrated the feasibility of the MR1-TCR in the engineered iPSCs, which also express CD19 CAR and hnCD16. Upon T cell differentiation, the engineered iPSC-derived T cells could recognize multiple hematological and solid tumor cell lines. Expression of hnCD16 also enhanced killing of CD20^+^ Raji cells when combined with Rituximab or HER2^+^ SKOV3 cells in the presence of anti-HER2 monoclonal antibody (Herceptin). Besides, the CD19 CAR T cells expressing either MR1-TCR or hnCD16 could eliminate CD19-negative lymphoma cells in the co-culture system (129). Altogether, these studies demonstrate the feasibility of iPSCs as a potential renewable cell source of CAR T cells and pave the way for developing off-the-shelf CAR T cell products with enhanced therapeutic efficacy (**Figure 3**).

## Challenges and Future Perspectives

Adoptive immunotherapy using CAR T cells has shown great success in patients with relapse and refractory B cell malignancies. While autologous T cells provide safety regarding lower risks of adverse side effects such as GvHD, the manufacturing process takes too long for some patients. In addition, the T cell doses largely depend on each individual. This becomes challenging in patients with a low number of T cells. *Ex vivo* expansion of T cells can result in T cell exhaustion, which reduces effector functions. These issues limit the clinical utility. Recently, the treatment using allogeneic T cells from healthy donors has gained more interest since the cells can be prepared and comprehensively validated in advance as off-the-shelf cell products, which can eventually lower the manufacturing cost and time (130). Advances in genome editing technologies have generated various types of engineered T cells with enhanced antigen specificity and persistence, and reduced alloreactivity so the cells can be applied to patients with broader histocompatibility. At present, several clinical trials are being performed to test the safety and efficacy of these engineered T cells, as reviewed in (131).

Meanwhile, iPSCs have been used as a starting cell source for the generation of immune cells for next-generation adoptive immunotherapy. The iPSCs offer advantages such as unlimited proliferation and the ability to differentiate into various cell types, including T cells and NK cells, and ease of multiplexed genome editing. With these properties, the engineered iPSC clones can be isolated, expanded, differentiated, functionally validated and banked in advance (132). However, there are several manufacturing and regulatory hurdles that need to be overcome. For example, the reprogramming methods must be integration-free to avoid potential mutagenesis and transgene reactivation. The process must be performed under cGMP standards (133). At the Center for iPS Cell Research and Application (CiRA), Kyoto University, Japan, the clinical-grade clonal master cell banks were derived from peripheral blood or umbilical cord blood of HLA-homozygous healthy volunteers using episomal plasmid reprogramming (134). Before the secondary cell stock can be used, it is essential to ensure that the cells exhibit normal karyotype and the residual plasmids were absent. Genomic integrity associated with reprogramming and prolonged culture of the established iPSC line, such as chromosomal alterations, copy number variations (CNV), and indel mutations, should be determined using whole-exome sequencing and SNP array, or whole-genome sequencing (134, 135). In addition, if the iPSCs are genetically engineered using the CRISPR/Cas9 system, the off-target activity from the incorrect binding of sgRNA can often occur and result in insertion-deletion (indel) mutations. Therefore, after clonal selection, it is recommended to conduct the whole-genome sequencing and careful screening of the clones for sterility, mycoplasma, and endotoxin before they are applied in clinics (136).

Apart from the quality control of the established iPSC line, the quality control of the final product, in this case, differentiated T cells, must be performed to evaluate the phenotype and function both *in vitro* and in pre-clinical studies. The differentiation protocol to generate T cells should be developed under a xenogeneic-free system i.e., without serum supplementation or mouse stromal cells as supportive feeders. To date, most published protocols still rely on the use of xenogeneic feeder cells. Although a recent study reported the use of the immobilized-DL-4 protein to generate clinically relevant functional iPSC-derived CD8αβ^+^ CAR-T cells (iCART), the therapeutic efficacy of iCART cells was more inferior than that of primary CART cells. This was due to the absence of CD4^+^ T cells, which also play an important role in the anti-tumor effect of CAR T cell therapy (75). While the 3D ATO platform could produce CD4^+^ T cells, this approach still requires co-culture with the mouse MS5-DLL4 cell line (137). Therefore, the generation of clinical-scale iPSC-derived functional T cells consisting of both CD8^+^ and CD4^+^ cells is necessary (138). Furthermore, the risk of tumor formation after transplantation due to residual pluripotent cells is the most significant concern. Cell sorting should be done to eliminate the contaminating cells as part of a quality check. In addition, the tumorigenicity test using immunodeficient mice such as NOG mice is also required to ensure that the transplanted cells are safe for clinical translation (133, 139). It is worth noting that the cell manufacturing process is far more sophisticated and complicated than pharmaceutical products. Altogether, these challenges are the main hurdles that slow down the clinical translation of iPSC-derived cell products.

As mentioned earlier, genome editing technology has been applied to generate universal iPSC-derived T cells. The removal of HLA-I can pose a potential safety risk. If the transplanted cells are virally infected or transformed into a tumor, they would not be recognized by the immune cells. Therefore, the solution to these problems is to introduce a suicide gene such as inducible Caspase 9 (iCas9) into the cells. Upon activation by a specific chemical inducer of dimerization (CID), the caspase cascade is induced, and the cells rapidly undergo apoptosis (140). This suicide system was previously tested in the T-iPSCs, and the results showed that the cytotoxic T cells derived from the iC9-expressing T-iPSCs were effective against EBV-induced tumors in the mouse model. Upon administration with CID, the iC9 system was activated, leading to apoptosis of CTLs. The suicide system can also be exploited to eliminate contaminating iPSCs or tumors derived from iPSCs as well as preventing adverse events such as GvHD, cytokine release syndrome, “on-target, off-tumor toxicities” in iPSC-derived T cell therapy (82).

Other concerns observed in CAR T cell therapy could also be considered for developing iPSC-derived T cells. The therapeutic efficacy of CAR T cell therapy mainly depends on the identification of the tumor-associated antigens or neoantigens that are expressed only on the tumor cells and not on the healthy cells. The ideal target antigen will have fewer adverse effects from “on-target, off-tumor toxicities” (141). Furthermore, in solid tumors, the immunosuppressive tumor microenvironments (TME) represent a significant barrier that impairs the function of CAR T cells. Several approaches have been applied to alter the TME from immunosuppressive to pro-inflammatory, including the use of a conditioning regimen prior to T cell infusion, small molecules to interfere with immunosuppressive cells, and blocking antibodies such as anti-PD-1 scFv to inhibit immune checkpoints (142, 143) as well as engineering CAR to express cytokine receptor or to secrete cytokines such as IL-12, IL-18, IL-15 to increase T cell persistence and anti-tumor efficacy (141, 144–147). To date, the CAR T cell therapy for solid tumors in clinical trials has not been effective since T cells cannot penetrate and survive in the TME. To overcome these hurdles, CAR platforms in other immune cells have been explored. One of which is macrophages that have abilities to penetrate the TME, perform phagocytosis and antigen presentation, and interact with other immune cells in the TME. Recently, Zhang et al. incorporated CD19-specific CAR into iPSCs and differentiated them into macrophages (CAR-iMac). Upon activation with leukemia and lymphoma cells, the CAR-iMAC were polarized toward the pro-inflammatory M1 subtype and able to phagocytose the tumor cells in an antigen-dependent manner. Therefore, combining iPSC-derived CAR T cells and CAR-iMac may provide an improved outcome in patients with the heavy burden of solid tumors (148).

## Conclusion

Advances in iPSC and genome editing technologies offer great promise toward the next-generation ACT where the iPSCs can be engineered to have a more potent cytotoxic function, increased persistence, and less immunogenicity. The iPSC-derived CAR T cells can be prepared and validated in advance as off-the-shelf products to be administered to a large number of cancer patients. Although several hurdles and challenges remain to be overcome, this strategy will provide an infinite supply of true off-the-shelf cell products for cancer immunotherapy.

## Author Contributions

All authors contributed to the article and approved the submitted version.

## Funding

This study was supported by grants from the Thailand Research Fund (grant no. RSA6280090), the Program Management Unit for Human Resources & Institutional Development, Research and Innovation (grant no. B05F630080), the Siriraj Research Fund, Faculty of Medicine Siriraj Hospital, Mahidol University (grant number (IO) R016234002) and Mahidol University. RN is supported by the Development and Promotion of Science and Technology Talents Project. MW is supported by Chalermphrakiat Grant, Faculty of Medicine Siriraj Hospital, Mahidol University.

## Conflict of Interest

The authors declare that the research was conducted in the absence of any commercial or financial relationships that could be construed as a potential conflict of interest.

## Publisher’s Note

All claims expressed in this article are solely those of the authors and do not necessarily represent those of their affiliated organizations, or those of the publisher, the editors and the reviewers. Any product that may be evaluated in this article, or claim that may be made by its manufacturer, is not guaranteed or endorsed by the publisher.
